# Long-Term Rodent Surveillance after Outbreak of Hantavirus Infection, Yosemite National Park, California, USA, 2012

**DOI:** 10.3201/eid2603.191307

**Published:** 2020-03

**Authors:** Mary E. Danforth, Sharon Messenger, Danielle Buttke, Matthew Weinburke, George Carroll, Gregory Hacker, Michael Niemela, Elizabeth S. Andrews, Bryan T. Jackson, Vicki Kramer, Mark Novak

**Affiliations:** California Department of Public Health, Sacramento, California, USA (M.E. Danforth, G. Hacker, M. Niemela, E.S. Andrews, B.T. Jackson, V. Kramer, M. Novak);; California Department of Public Health, Richmond, California, USA (S. Messenger);; National Park Service, Fort Collins, Colorado, USA (D. Buttke);; National Park Service, El Portal, California USA (M. Weinburke, G. Carroll);

**Keywords:** hantavirus, Sin Nombre virus, viruses, hantavirus pulmonary syndrome, rodents, Peromyscus maniculatus, deer mice, Rodentia, infection, surveillance, parks, recreational, disease outbreaks, seroepidemiology, zoonoses, Yosemite National Park, California, United States

## Abstract

In 2012, a total of 9 cases of hantavirus infection occurred in overnight visitors to Yosemite Valley, Yosemite National Park, California, USA. In the 6 years after the initial outbreak investigation, the California Department of Public Health conducted 11 rodent trapping events in developed areas of Yosemite Valley and 6 in Tuolumne Meadows to monitor the relative abundance of deer mice (*Peromyscus maniculatus*) and seroprevalence of Sin Nombre orthohantavirus, the causative agent of hantavirus pulmonary syndrome. Deer mouse trap success in Yosemite Valley remained lower than that observed during the 2012 outbreak investigation. Seroprevalence of Sin Nombre orthohantavirus in deer mice during 2013–2018 was also lower than during the outbreak, but the difference was not statistically significant (p = 0.02). The decreased relative abundance of *Peromyscus* spp. mice in developed areas of Yosemite Valley after the outbreak is probably associated with increased rodent exclusion efforts and decreased peridomestic habitat.

A disease outbreak in North America caused by a hantavirus occurred in 1993 in the Four Corners area of the southwestern United States (*1*). Deer mice (*Peromyscus maniculatus*) were identified as the primary reservoir of Sin Nombre virus (SNV) (*2*), an orthohantavirus and the etiologic agent of hantavirus pulmonary syndrome (HPS) (*3*). That outbreak might have been associated with an El Niño weather event the preceding winter, which could have led to increases in deer mouse infestations in buildings (*4*). Investigations into the outbreak and subsequent HPS cases found most cases had probable indoor exposures (*5**,**6*) and almost one fourth of all human case exposures were associated with a recreational setting (*7*).

During the summer of 2012, a total of 10 persons subsequently given a diagnosis of hantavirus infection visited Yosemite National Park in California, USA (*8**,**9*). SNV exposure for 9 case-patients was associated with staying overnight in a signature tent cabin, a canvas tent structure with interior insulated walls, located in Curry Village in Yosemite Valley (*8**,**9*); the tenth infection was associated with lodging in regular tent cabins in the Tuolumne Meadows area. The subsequent environmental investigation found that most of the signature tent cabins had rodent infestations in the insulated walls. A high overnight trap success rate (51%) for *Peromyscus* spp. mice and a 14% (10/73) SNV seroprevalence in deer mice were observed in Curry Village during the initial trapping event in August 2012 (*8*). The park responded by closing and subsequently removing the signature tent cabins, increasing staff and visitor education for HPS prevention, enhancing mouse control measures in and around human-made structures (*8**,**9*), and applying rodent exclusion measures to other buildings (*8*). In September 2012, the *Peromyscus* spp. trap success rate in Curry Village was substantially lower (14%), and no (0/10) deer mice were positive for SNV (*8*).

We summarize rodent trappings and SNV serosurveys for *Peromyscus* mice in Yosemite Valley and Tuolumne Meadows after the outbreak of infection with hantavirus during 2012. These activities were conducted to monitor relative abundance of deer mice, help assess the peridomestic rodent control efforts in the park, and reduce HPS risk in this heavily used recreational area. We compared *Peromyscus* spp. mouse overnight trap success rates and captured *Peromyscus* mouse species composition and SNV seroprevalence in deer mice from peridomestic sites in Yosemite Valley during 2013–2018 with findings from the initial outbreak investigation in August–September 2012 and with findings of similar trapping events conducted in developed areas of Tuolumne Meadows. We also evaluated whether location or climatic factors influenced relative rodent abundance and SNV seroprevalence. Finally, we sought to identify demographic characteristics of SNV-positive deer mice captured in Yosemite National Park.

## Methods

### Site Selection, Description of Study Areas, and Study Period

**Figure 1:**
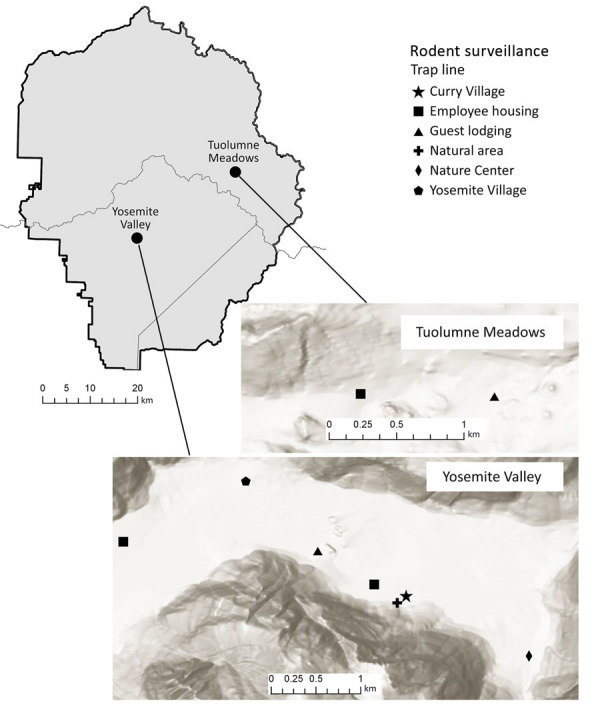
Yosemite National Park, California, USA, and trapping sites, with hillside shading, in Yosemite Valley and Tuolumne Meadows. Sources of mapping data were Esri (https://www.esri.com), Airbus Defence and Space, US Geological Survey, National Geospatial-Intelligence Agency, National Aeronautics and Space Administration, Consultative Group on International Agricultural Research, N. Robinson, **National Center for Ecological Analysis and Synthesis,** National Library Service, Ordnance Survey, National Mapping Association, Geodatastryelsen, Rijkswaterstaat, General Services Administration, Geoland, Federal Emergency Management Agency, Intermap, and the Geographic Information System user community.

Yosemite National Park is in the Sierra Nevada mountain range in California. Yosemite Valley (37.745570, –119.593604) is located in the west-central portion of the park (Figure 1) and covers ≈18 km^2^ at an average elevation of 1,209 m. The primary habitat of Yosemite Valley is lower montane forest, dominated by California black oak (*Quercus kelloggii*), ponderosa pine (*Pinus ponderosa*), incense cedar (*Calocedrus decurrens*), and white fir (*Abies concolor*) (*10*). Curry Village is located near the eastern end of Yosemite Valley in a highly developed area that contains other visitor lodging, park administration buildings, and staff housing.

During May 2013–October 2018, rodent surveillance by the California Department of Public Health (CDPH) Vector-Borne Disease Section was conducted 11 times at Curry Village and other nearby peridomestic sites on the basis of park staff requests (Figure 1). In 2013, trapping was conducted in Yosemite Valley only in May. In subsequent years, trapping was conducted twice annually, in spring (May–June), to assess peridomestic deer mouse abundance and identify potential problem areas before peak tourist visitation, and fall (October–November) (Table 1), when peridomestic deer mouse trap success typically peaks (CDPH and National Park Service [NPS], unpub. data).

**Table 1:** Dates, locations, and climate data for Sin Nombre virus surveillance, Yosemite National Park, California, USA, 2013–2018

Trap date	Location	Mean monthly temperature, °C			Total water year, mm	Total water year, mm, from year before
		Trapping month	6 months before	1 year before		
2013 May 30	Yosemite Valley	12.9	7.0	16.9	643.01	547.43
2013 Sep 10	Tuolumne Meadows	9.2	0.7	10.5	541.02	426.65
2014 Jun 26	Yosemite Valley	18.2	7.5	18.4	451.25	643.01
2014 Sep 9	Tuolumne Meadows	9.9	−0.8	9.2	384.94	541.02
2014 Nov 13	Yosemite Valley	7.6	18.3	7.5	451.25	643.01
2015 May 19	Yosemite Valley	11.2	7.6	13.2	398.61	451.25
2015 Aug 26	Tuolumne Meadows	12.6	1.4	11.4	316.54	384.94
2015 Oct 22	Yosemite Valley	13.7	8.8	14.6	398.61	451.25
2016 May 25	Yosemite Valley	11.7	4.1	11.2	956.50	398.61
2016 Jun 22	Tuolumne Meadows	10.6	−5.5	11.4	744.60	316.54
2016 Oct 12	Yosemite Valley	11.4	9.3	13.7	956.50	398.61
2017 May 24	Yosemite Valley	12.9	7.3	11.7	1,871.10	956.50
2017 Aug 9	Tuolumne Meadows	12.9	−0.9	12.5	1,637.41	744.60
2017 Oct 18	Yosemite Valley	12.7	7.8	11.4	1,871.10	956.50
2018 May 17	Yosemite Valley	12.6	7.6	12.9	731.19	1,871.10
2018 Aug 22	Tuolumne Meadows	12.8	−3.7	12.9	648.49	1,637.41
2018 Oct 9	Yosemite Valley	12.0	9.0	12.7	731.19	1,871.10

Six additional rodent trapping events were conducted annually during 2013–2018 in developed areas of Tuolumne Meadows (37.873107, −119.435709), where previous HPS case-patients have been exposed (*8*); Tuolumne Meadows is located ≈26 km northeast of Yosemite Valley (Figure 1) at an elevation of 2,602 m. The primary habitat is upper montane-subalpine, dominated by Sierra lodgepole (*Pinus contorta murryana*), Ross sedge (*Carex rossii*), western white pine (*Pinus monticola*), and mountain hemlock (*Tsuga mertensiana*) (*10*). Trapping in Tuolumne Meadows was conducted in and around guest lodging and employee housing (Figure 1). With the exception of June 2016, trapping events in Tuolumne Meadows were conducted in August or September, months when this area was most likely to be accessible and facilities open.

### Trapping Protocol

All rodent trapping and handling was conducted according to protocols of the CDPH Institutional Animal Care and Use Committee (ACUP no. 2013-14–no. 2018-14). Rodent trapping used Sherman live traps (H.B. Sherman Traps, https://www.shermantraps.com). Each event consisted of a single overnight trapping period, with 100–200 traps in Yosemite Valley or 44–180 traps in Tuolumne Meadows. Traps were primarily placed outside buildings and tent cabins and left open from ≈5:00 pm to 8:00 am the following day. A total of 75–81 traps were set outdoors in Curry Village during each Yosemite Valley trapping event. A limited number of traps (0–26/event) were placed indoors to evaluate potential for rodent ingress or in response to reported mouse activity. Beginning in November 2014, a total of 25 traps/event were also set on a transect through a natural habitat adjacent to Curry Village, 25–75 m from any human-made structure, for comparison to peridomestic locations.

Traps were baited with a mixture of corn, oats, and barley, and a ball of polystyrene fill was placed inside as nesting material. Captured rodents were anesthetized with isoflurane, identified to species, sex, and age group, measured for weight, and assessed for the presence of ear scars or notches. Approximately 100 µL of blood was collected into a heparinized capillary tube from the retro-orbital sinus, then stored on ice or refrigerated before transport to the laboratory. All *Peromyscus* mice collected in or near a building were humanely euthanized. *P. boylii* (brush mice) trapped in the natural area adjacent to Curry Village and all other rodent species trapped were released at their point of capture.

### Sample Testing

*Peromyscus* mouse blood samples were submitted to the CDPH Viral and Rickettsial Disease Laboratory to screen for evidence of antibodies against SNV and SNV RNA. Serum or whole blood was analyzed for SNV IgG by using an ELISA (*2*) to detect antibody directed against a purified recombinant Sin Nombre nucleocapsid protein that is strongly recognized by antibodies against orthohantaviruses associated with subfamily *Sigmondontinae* rodents. Rodent blood samples were also screened for evidence of SNV RNA by using a real-time reverse transcription PCR (RT-PCR) targeting an 81-nt region of the small segment of the genome (GenBank accession no. L33816) (*11*), which is highly conserved across known SNV strains.

### Data Analysis

We excluded all non-*Peromyscus* rodent captures from data analyses. We estimated relative *Peromyscus* rodent abundance by using trap success (no. *Peromyscus* rodents trapped/no. traps set) for each trapping event at each site type and calculated the proportion of captured *Peromyscus* mice that were deer mice for each site type at each trapping event (no. deer mice/no. all *Peromyscus* rodent captures). We estimated seroprevalence by the percentage of deer mice sampled that were positive for SNV antibodies. We obtained climate data from the PRISM climate group (*12*); variables used were mean monthly temperature for the month of the trapping event (°C), mean monthly temperature from 6 months before (°C), mean monthly temperature from 12 months before (°C), current water year (total precipitation from the preceding October 1 through April 30, mm), and the previous water year (Table 1).

We analyzed data by using R statistical software (*13*). We made comparisons for trap success, proportions of *Peromyscus* species captures that were deer mice, and deer mouse seroprevalence in Yosemite Valley during June 2013–October 2018 to those from August 2012 and September 2012 in Yosemite Valley, in the natural area adjacent to Curry Village (2014–2018 for both datasets), and in Tuolumne Meadows by using χ^2^ analysis. Because we made 5 comparisons, we applied the Bonferroni correction to χ^2^ analyses; only p<0.01 was considered significant. If a cell size was <5 by any χ^2^ analysis, we then used the Fisher exact test.

We conducted regression analyses on all data collected for Yosemite Valley and Tuolumne Meadows after 2013. We used multivariate linear regression to find associations between relative rodent abundance and time as a continuous variable, season as a categorical variable (spring or fall), site (Curry Village, Curry Village natural area, other Yosemite Valley peridomestic sites, and Tuolumne Meadows), current and previous climatic variables, and current and previous relative rodent abundance. We also used multivariate linear regression to determine whether the proportion of *Peromyscus* captures at an event that were deer mice was associated with time, season, site, current and previous climatic variables, and current and previous proportions of deer mouse captures. We analyzed the relationship between seroprevalence and date, season, peridomestic versus natural area, current and previous relative rodent abundance and dominance by using multivariate linear regression. We then used multivariate logistic regression to identify which demographic variables (age, sex, weight, presence of ear notches/scars) were associated with detecting positive deer mice.

## Results

### Summary Statistics

During May 2013–October 2018, CDPH conducted 11 trapping events (1,574 trap nights of surveillance) at peridomestic sites in Yosemite Valley, and captured 231 rodents (overall trap success rate 14.7%); there were no recaptures. Thirty-one (2.0%) traps were set inside buildings, and only 1 deer mouse was captured indoors (trap success rate 3.2%). Deer mice represented 148 (64.1%) of all captures; the remainder consisted of 70 (30.3%) brush mice, 2 shrews (*Sorex* spp.), 5 house mice (*Mus musculus*), 2 roof rats (*Rattus rattus*), and 4 unidentified *Peromyscus* rodents that escaped before processing. 

**Figure 2:**
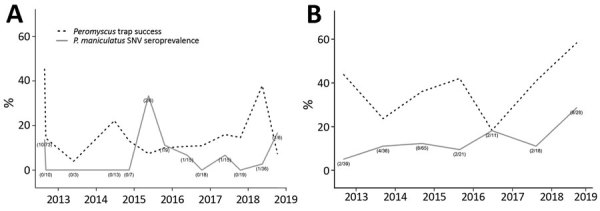
*Peromyscus* rodent trap success and seroprevalence (with sample sizes) of SNV in deer mice (*Peromyscus maniculatus*), Yosemite National Park, California, USA, 2012–2018. A) Yosemite Valley; B) Tuolumne Meadows. Numbers in parentheses indicate no. positive deer mice/no. tested. Figures include data from the August and September 2012 outbreak investigation (*8*) for reference. SNV, Sin Nombre virus.

Blood samples were collected from 147 deer mice (Table 2), and 7 (4.8%) were positive for antibodies to SNV (Figure 2, panel A). We also tested 67 brush mice blood samples for SNV antibodies, and all were negative. We retested 6 deer mouse blood samples that had SNV antibodies and 131 that did not have SNV antibodies for SNV RNA by real-time RT-PCR. Three (50%) of the 6 antibody-positive deer mice were also positive for SNV RNA, and only 1 (0.8%) of 131 seronegative deer mice showed positive results. We also tested 34 brush mice by using real-time RT-PCR; all were negative for SNV RNA.

**Table 2:** Characteristics of *Peromyscus maniculatus* rodents tested for Sin Nombre virus in Yosemite National Park, California, USA, 2013–2018*

Characteristic category	Value
Sex	
M	21/147 (14.3)†
F	12/186 (6.5)
Age	
Adult	24/189 (12.7)
Subadult	9/107 (8.4)
Juvenile	0/37 (0)
Ear scarred, torn, or notched‡	3/13 (18.8)
Location	
Curry Village	4/88 (4.5)
Other Yosemite Valley periodomestic area	3/59 (5.1)
Curry Village natural area	0/7 (0)
Tuolumne Meadows	26/179 (14.5)†
Mean weight, g	
Antibody negative	
M	14.7
F	15.9
Antibody positive	
M	17.1§
F	17.2§

*Values are no. antibody positive/no. tested (%) unless otherwise indicated. †Significantly greater than others in category. ‡Observations about the presence of ear scars, tears, or notches were not systematically recorded. §Significant difference between antibody positive and antibody negative.

The natural area adjacent to Curry Village was trapped during 9 events from November 2014 through October 2018 for 223 trap nights. Thirty-three *Peromyscus* mice and 1 shrew were captured (overall trap success rate 15.2%). One brush mouse was captured twice. Deer mice represented 7 (20.6%) of the captures; 26 (76.5%) of captures were brush mice. We tested blood samples from 7 deer mice (Table 2) and 13 brush mice for antibodies to SNV; all were negative. We retested 6 deer mice and all 13 brush mice by using real-time RT-PCR; all were negative for SNV RNA.

During September 2013–October 2018, a total of 534 trap nights during 6 surveillance events in Tuolumne Meadows captured 195 rodents (trap success rate 36.5%); there were no recaptures. Deer mice represented 179 captures (91.8%), and the remaining 16 captures consisted of 8 wood rats (*Neotoma cinerea*), 3 chipmunks (*Tamias* spp.), 4 golden-mantled ground squirrels (*Callospermophilus lateralis*), and 1 long-tailed vole (*Microtus longicaudus*). Blood samples were collected from all 179 deer mice (Table 2) and 26 (14.5%) were positive for SNV antibodies (Figure 2, panel B).

### Trends in *Peromyscus* spp. Trap Success

The overall *Peromyscus* trap success rate at peridomestic sites in Yosemite Valley (14.1%) (Figure 2, panel A) was significantly lower during 2013–2018 than that during the initial August 2012 outbreak investigation (χ^2^ 142.6; p<0.01), although not from the September 2012 surveillance event (χ^2^ <0.1; p = 0.99). Within Yosemite Valley, we found no significant difference in the *Peromyscus* trap success between Curry Village (14.2%) and other peridomestic sites in Yosemite Valley (13.9%; χ^2^ <0.1; p = 0.88). We also noted no significant difference in trap success rates between all Yosemite Valley peridomestic locations combined and the natural area adjacent to Curry Village (14.8%; χ^2^ 0.1; p = 0.78). However, *Peromyscus* trap success in Yosemite Valley during the study was significantly lower than that in Tuolumne Meadows (33.7%; χ^2^ 11.8; p<0.01) (Figure 2, panel B). When we performed analysis by using by multivariate regression, we found no significant association between relative rodent abundance and date, season, and any current or previous climatic variable.

### Trends in *Peromyscus* spp. Rodent Captures

**Figure 3:**
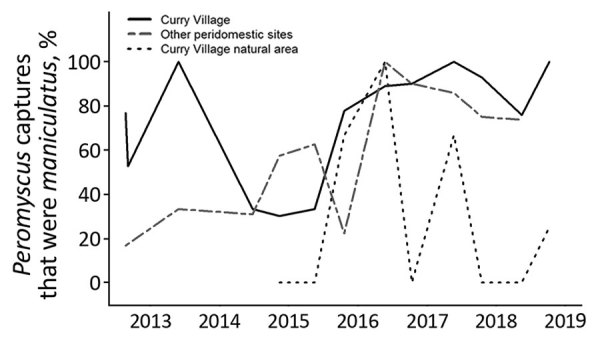
Proportion of *Peromyscus* rodent captures that were *P.* *maniculatus* from areas of Yosemite Valley, Yosemite National Park, California, USA, 2012–2018. Figure includes data from the August– September 2012 outbreak investigation (*8*) for reference. SNV, Sin Nombre virus.

The proportion of *Peromyscus* spp. rodent captures that were *P. maniculatus* deer mice (82.2%) at peridomestic locations in Yosemite Valley during 2013–2018 was not different from those observed in August 2012 (73.3%; χ^2^ 3.2; p = 0.07) and September 2012 (52.6%; χ^2^ 1.8; p = 0.18) (Figure 3). Although most *Peromyscus* rodent captures at peridomestic sites in Yosemite Valley were deer mice (75.4%), deer mice were significantly less likely to be trapped in the natural area adjacent to Curry Village (24.2%; χ^2^ 33.1; p<0.01). The proportion of *Peromyscus* rodent captures that were deer mice did not have a significant linear relationship with time, season, site, current and previous climate variables, or current and previous trap success.

### Trends in SNV Seroprevalence

SNV antibody seroprevalence in deer mice sampled at peridomestic sites in Yosemite Valley was not significantly different than that observed during August 2012 (χ^2^ 5.4; p = 0.02) or September 2012 (p = 1.00 by Fisher exact test). Within Yosemite Valley, no significant difference occurred in detection of SNV-positive deer mice in peridomestic areas compared with the natural area (p = 1.00 by Fisher exact test). However, seroprevalence was significantly lower at peridomestic sites in Yosemite Valley than in Tuolumne Meadows during the study (χ^2^ 32.9; p<0.01). We found no relationship between seroprevalence in Yosemite Valley deer mice and time, season, peridomestic versus natural area, or concurrent or previous relative *Peromyscus* rodent abundance and *P. maniculatus* mouse dominance. When analyzed by logistic regression, we found that seropositive deer mice from Yosemite Valley and Tuolumne Meadows were significantly more likely to be male (β = 1.09; p = 0.02) and have higher body weights (β = 0.16; p = 0.01); no other demographic variables were significant.

## Discussion

During the initial Yosemite hantavirus outbreak investigation in August 2012, a robust population of deer mice in Curry Village was identified, although SNV seroprevalence was not unusually increased (*8*). Just a few weeks later, after the signature tent cabins were closed and rodent control and exclusion measures were enacted, trap success for deer mice was substantially lower (*8*). Our study found that overall trap success during May 2013–October 2018 in Yosemite Valley remained lower than that observed during August 2012. The rodent control measures implemented by the park and concessionaires have likely contributed to lower *Peromyscus* rodent trap success in these peridomestic locations. To a lesser degree, the cumulative effect of removing *Peromyscus* mice from peridomestic locations during our surveillance events might have also contributed to the control effort. Although current and previous precipitation amounts were not associated with *Peromyscus* rodent trap success, we cannot rule out the effects of the historic drought in California during 2011–2015 (*12*). The end of the drought might have contributed to the trend of increasing trap success rates seen during 2016–2018. Although a few SNV-seropositive deer mice have continued to be detected since 2012, rodent control measures that limit the number of deer mice around and in buildings have likely decreased HPS exposure risk (*14**,**15*).

Overall, deer mice represented a similar proportion of the *Peromyscus* rodent captures from peridomestic locations during 2013–2018 as during the outbreak investigation in 2012. However, the proportion of deer mouse and brush mouse captures from these locations fluctuated after 2012, suggesting the relative abundance of these species changed over time. Interspecific competition between these sympatric species (*16*), climatic factors, or some combination of these effects probably contributed to the observed trends in trap success, but habitat preferences might also affect local abundance. Deer mice are the most common *Peromyscus* species in California, found in almost any habitat, and commonly enter buildings (*17*). Brush mice are found mainly below an elevation of 2,000 m (*17*) and have a preference for rocky areas in brush or woodlands (*18*), although they will readily enter human-made structures. Although preferred habitats for both species occur in Yosemite Valley, highly developed locations providing human-made harborage and food sources might favor deer mouse abundance.

Although deer mice predominated at peridomestic sites, brush mice were captured more frequently at the natural area sampled. This trap line was only 25–75 m from tent cabins and other buildings in Curry Village, both locations potentially within typical home ranges of deer mice (41–4,452 m^2^) and brush mice (162–3,845 m^2^) (*19*). Despite the proximity of these habitats, brush mice were the dominant, often only, *Peromyscus* species trapped in the natural area. This finding supports the need for minimizing peridomestic harborage that might favor deer mouse abundance. In addition, maintaining natural environments to the extent possible in Yosemite Valley could increase competition from brush mice, which are not known reservoirs of SNV (*20*). Increasing rodent diversity could also reduce SNV prevalence in deer mice (*21**,**22*).

We were unable to detect many major trends in deer mouse seroprevalence. Although SNV seroprevalence in Yosemite Valley decreased during August–September 2012 and typically remained lower in subsequent years, we found no significant differences in seroprevalence between either month during 2012 and that observed during 2013–2018 because of Bonferroni adjustment for multiple comparisons and low p value threshold. Other, much larger, studies have detected relationships between deer mouse seroprevalence and previous rodent population density (*14**,**23**,**24*) or age (*14**,**21**,**23**,**25**,**26*), neither of which we observed, probably because of our smaller sample size. Also, potentially because of inconsistent collection of qualitative observations of body condition, we were unable to determine whether seropositive deer mice were more likely to have wounds (*14**,**23**,**25**–**27*). However, we did find that male and heavier deer mice were more likely to be seropositive, as seen in other studies (*14**,**21**,**23**,**25**–**27*).

We also compared trapping results and SNV seroprevalence from Yosemite Valley during 2013–2018 to Tuolumne Meadows. Despite trapping around similar types of buildings, the deer mouse trap success rate and SNV seroprevalence were higher in Tuolumne Meadows. This location is 1,400 m higher in elevation, outside the range of brush mice, and no other *Peromyscus* rodent species have been trapped here during previous CDPH surveillance events (CDPH, unpub. data). Tuolumne Meadows is also less developed than Yosemite Valley, and most buildings are used only seasonally, typically during June–September. Given the absence of other *Peromyscus* rodent species and abundance of seasonally used buildings in an otherwise natural montane habitat, the consistent abundance of deer mice and higher SNV seroprevalence at Tuolumne Meadows is not surprising. Higher SNV seroprevalence rates relative to Yosemite Valley were observed in previous surveillance events in this area (CDPH, unpub. data) and in deer mice sampled at other higher elevations in California (*28*). This area was associated with 3 previous HPS cases during 2000, 2010, and 2012 (*8*), and although more cases of infection with hantavirus have been associated with Yosemite Valley, all 9 cases were linked with the 2012 outbreak and the subsequently removed signature tent cabins. Our surveillance results and the sporadic occurrence of HPS cases underscore the need for maintaining hantavirus awareness and prevention measures in the Tuolumne Meadows area.

Since 2012, the NPS and concessionaires have expanded their efforts beyond Curry Village to improve rodent exclusion in other buildings, reduce rodent harborage in peridomestic habitats, and conduct regular mouse trapping in developed areas of the park (*8*). A previous study in Yosemite found that rodent-proofed homes are less likely to be infested with mice and, if infested, have fewer mice (*29*). In addition to snap-trapping indoors, Yosemite staff conduct routine outdoor snap-trapping around buildings that are difficult to exclude, which assists in peridomestic rodent control and provides monitoring for spatiotemporal increases in *Peromyscus* rodent abundance. Early indications of increases in rodent abundance prompt the initiation of specified actions to reduce human risk for exposure to SNV (*30*). To assist the park and concessionaire with identifying rodent exclusion issues, CDPH has conducted >300 building evaluations during 2013–2018.

After the outbreak during 2012, NPS and concessionaires expanded their public education programs to reduce the risk for HPS. NPS added hantavirus information to its Yosemite website, placed educational posters in central locations, and offers informational brochures to visitors (*8*). Visitors at Curry Village and other tent cabin lodgings are provided with information about hantavirus at check-in and prevention methods are posted in each tent cabin (*8*). These efforts, combined with improvements in rodent exclusion and control measures and ongoing rodent surveillance, have helped to strongly reduce peridomestic abundance of deer mice and the risk for exposure to HPS for visitors and staff in Yosemite.
